# Early auditory responses to speech sounds in Parkinson’s disease: preliminary data

**DOI:** 10.1038/s41598-022-05128-8

**Published:** 2022-01-19

**Authors:** Fatemeh Mollaei, Douglas M. Shiller, Shari R. Baum, Vincent L. Gracco

**Affiliations:** 1 School of Psychology and Clinical Language Sciences, University of Reading, Early Gate, Whiteknights, Reading, Berkshire, RG6 6ES UK; 2 Centre for Research on Brain, Language and Music, 3640 Rue de la Montagne, Montreal, QC H3G 2A8 Canada; 3 School of Communication Sciences and Disorders, McGill University, 2001 McGill College Avenue, Montreal, QC H3A 1G1 Canada; 4 École d’orthophonie et d’audiologie, Université de Montréal, 7077 Avenue du Parc, Local 3001-31, Montreal, QC H3C 3J7 Canada; 5 CHU Sainte-Justine Research Centre, 5200 Bélanger East, Local GR-103, Montreal, QC H1T 1C9 Canada; 6 Haskins Laboratories, 300 George Street, New Haven, CT 06511 USA

**Keywords:** Sensorimotor processing, Diseases, Parkinson's disease, Basal ganglia, Auditory system

## Abstract

Parkinson’s disease (PD), as a manifestation of basal ganglia dysfunction, is associated with a number of speech deficits, including reduced voice modulation and vocal output. Interestingly, previous work has shown that participants with PD show an increased feedback-driven motor response to unexpected fundamental frequency perturbations during speech production, and a heightened ability to detect differences in vocal pitch relative to control participants. Here, we explored one possible contributor to these enhanced responses. We recorded the frequency-following auditory brainstem response (FFR) to repetitions of the speech syllable [da] in PD and control participants. Participants with PD displayed a larger amplitude FFR related to the fundamental frequency of speech stimuli relative to the control group. The current preliminary results suggest the dysfunction of the basal ganglia in PD contributes to the early stage of auditory processing and may reflect one component of a broader sensorimotor processing impairment associated with the disease.

## Introduction

Parkinson’s disease (PD), a manifestation of basal ganglia (BG) dysfunction, is associated with a number of speech production deficits in prosody, phonation and articulation, with phonation and laryngeal deficits the most prominent^1–5^. In addition to speech motor symptoms, auditory perceptual deficits ranging from self-monitoring to discrimination have been reported in PD^5–7^. Interestingly, it is during the monitoring of their own speech that PD participants often show the greatest differences from unimpaired speakers^3,8,9^. A common interpretation is that when individuals with PD are asked to produce speech with normal loudness (as judged by a speech-language pathologist), they perceive themselves as shouting or producing abnormally loud speech^8^. In addition, while listening at a given distance from a loudspeaker, individuals with PD estimated the loudness level to be significantly greater than that estimated by healthy control participants^2^.

These perceptual/sensory differences and their relationship with the speech motor output deficits of PD have been examined in several recent studies. In response to alterations in auditory feedback during speech, PD participants display an interesting characteristic. When faced with feedback consistent with a misplaced tongue position for a specific vowel (change in first formant frequency of vowel), PD participants exhibit reduced compensation compared to age-matched control participants^10^, consistent with a weaker motor response. In contrast, when faced with feedback consistent with a change in fundamental frequency (*f*_*o*_, voice pitch), PD participants exhibit increased compensation to the feedback shift^10–13^. An increased response to voice pitch shifts has also been observed in participants with Alzheimer’s disease^14,15^ and cerebellar degeneration^16^. In a recent study, we demonstrated that the increased response to a change in voice pitch is accompanied also by increased sensitivity in detecting pitch alterations during auditory feedback monitoring in participants with PD^4^. Pitch feedback manipulations were presented under conditions of production and listening. In the production condition, participants’ vocal pitch was shifted, and participants judged whether their speech output had been manipulated in real-time; participants’ responses to pitch shift change were simultaneously recorded. During the listening task, participants judged whether paired tokens of their previously recorded speech samples were the same or different. Under the production condition, the ability of participants with PD to identify the pitch shift was greater than that of the controls, with a trend for better detection during the listening condition^4^. Interestingly, in a parallel experiment, detection accuracy of first formant shifts was reduced in individuals with PD only during the listening condition^4^.

At the neural level, electrophysiological recordings in response to voice pitch shifts yielded larger event related potentials in the inferior frontal gyrus, precentral gyrus, postcentral gyrus, and middle temporal gyrus in PD participants^12^. While the results suggest cortical involvement in the enhanced pitch shift response, there are also reports in the literature to suggest involvement at the level of the brainstem for auditory processing in PD participants^17–19^. The brainstem, including the cochlear nuclei, the superior olivary complex, and the inferior colliculus of the midbrain comprise the auditory pathway to auditory cortical areas, with processing at each level^20^. The main focus of the current investigation was to evaluate the brainstem involvement in the processing of complex speech sounds in relation to the speech motor deficits in PD.

The auditory brainstem response (ABR) is an auditory evoked neural potential that provides a window into top–down and bottom–up processing of sensory information through the efferent and afferent projections of cortex, brainstem, and BG. Scalp-recorded auditory brainstem responses to complex sounds such as vowels include transient and sustained components that represent certain critical acoustic properties of speech stimuli^21^. Some of the acoustic properties of speech including pitch and formant frequencies are closely reflected within a component of the ABR (sustained response) known as the frequency-following response (FFR). The FFR reflects sustained electrical potentials that are precisely phase-locked to neuronal firing with an upper limit of about 1000 Hz in response to low to middle frequency periodic acoustic stimuli. Consequently, the FFR demonstrates a robust representation of time-varying *f*_*o*_ and harmonics corresponding to the pitch and first formant frequency (*F*_1_) of the vowel (^21,22^ for a review refer to^23^). Measures that are derived from the FFR response, such as amplitude and latency, represent a mapping between the auditory stimulus and the neural activity, which may be modified due to changes associated with disease (e.g., autism spectrum disorders, and mild cognitive impairment^24,25^) or exposure to auditory stimulation (e.g., music, and bilingualism^26,27^). Specific regions in the BG and thalamic nuclei, including the subparafascicular thalamic nucleus, send dopaminergic projections to the main auditory midbrain nucleus and the inferior colliculus, consistent with a modulatory role of the BG in auditory processing^28–31^. Based on these considerations, it is suggested that a dysfunctional dopaminergic system may contribute to differences in the processing of pitch and formant information for speech and ultimately to differences in speech motor output.

Here, we recorded FFR from the left mastoid during listening to a speech syllable. We compared patterns of phase-locking in individuals with PD and control participants on the assumption that it indexes—not solely, but to a large extent—subcortical interactions. On the basis of prior behavioral and perceptual results, we hypothesized that individuals with PD would show an increased FFR response only in the frequency range associated with *f*_*o*_ compared to control participants, consistent with a brainstem contribution to their speech perceptual and production impairment.

## Results

### Electrophysiology

**Figure 1:**
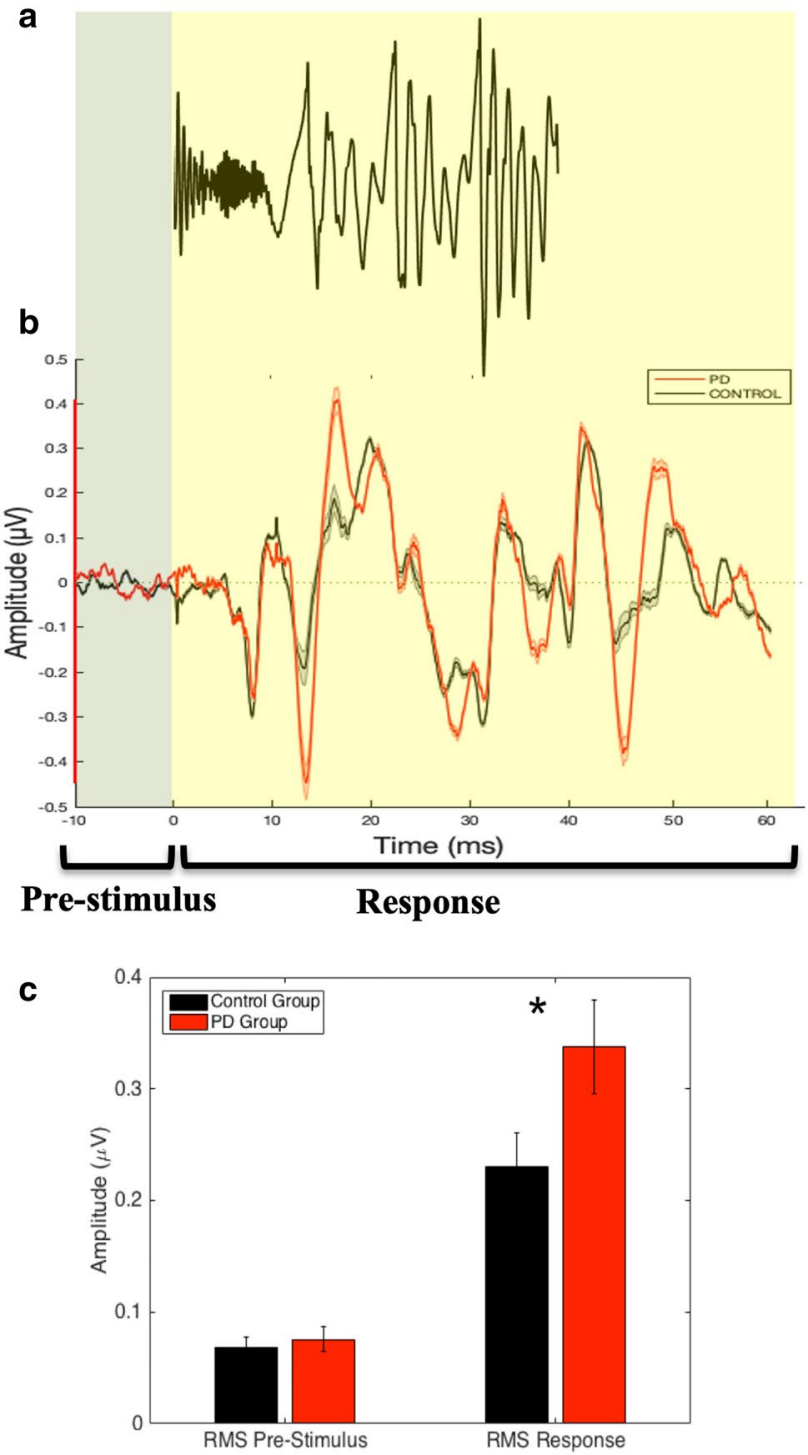
(**a**) Stimulus waveform. (**b**) Average waveform brainstem response to [da] in individuals with PD (red) and age- and gender-matched controls (black). The pre-stimulus period (− 10 to 0 ms) and the response period (0 to 60 ms) are shown. (**c**) Bar graph demonstrating between-group differences in RMS amplitude for the pre-stimulus and response periods. The error and shaded error bars represent the standard error. *Note*: *p < 0.01.

A multivariate analysis of variance (MANOVA) was used to compare root mean square (RMS) magnitudes (pre-stimulus and response) and spectral response amplitudes of the FFR (*f*_*o*_ and *F*_1_) between groups (PD vs. control). A waveform depicting the characteristics of the input stimulus [da], along with averaged FFR waveforms for the PD and control groups, is displayed in Fig. 1a and b. The Figure also includes a bar graph comparing the magnitudes of response RMS amplitudes during the pre-stimulus and stimulus periods, and it is displayed in Fig. 1c. In the time domain, no significant difference was observed between groups for the amplitude of the pre-stimulus region [*F*(1,28) = 0.24, *p* = 0.626]. In contrast, individuals with PD showed greater RMS amplitude for the frequency-following response [*F*(1,28) = 9.04, *p* = 0.006, η_p_^2^ = 0.244].

**Figure 2:**
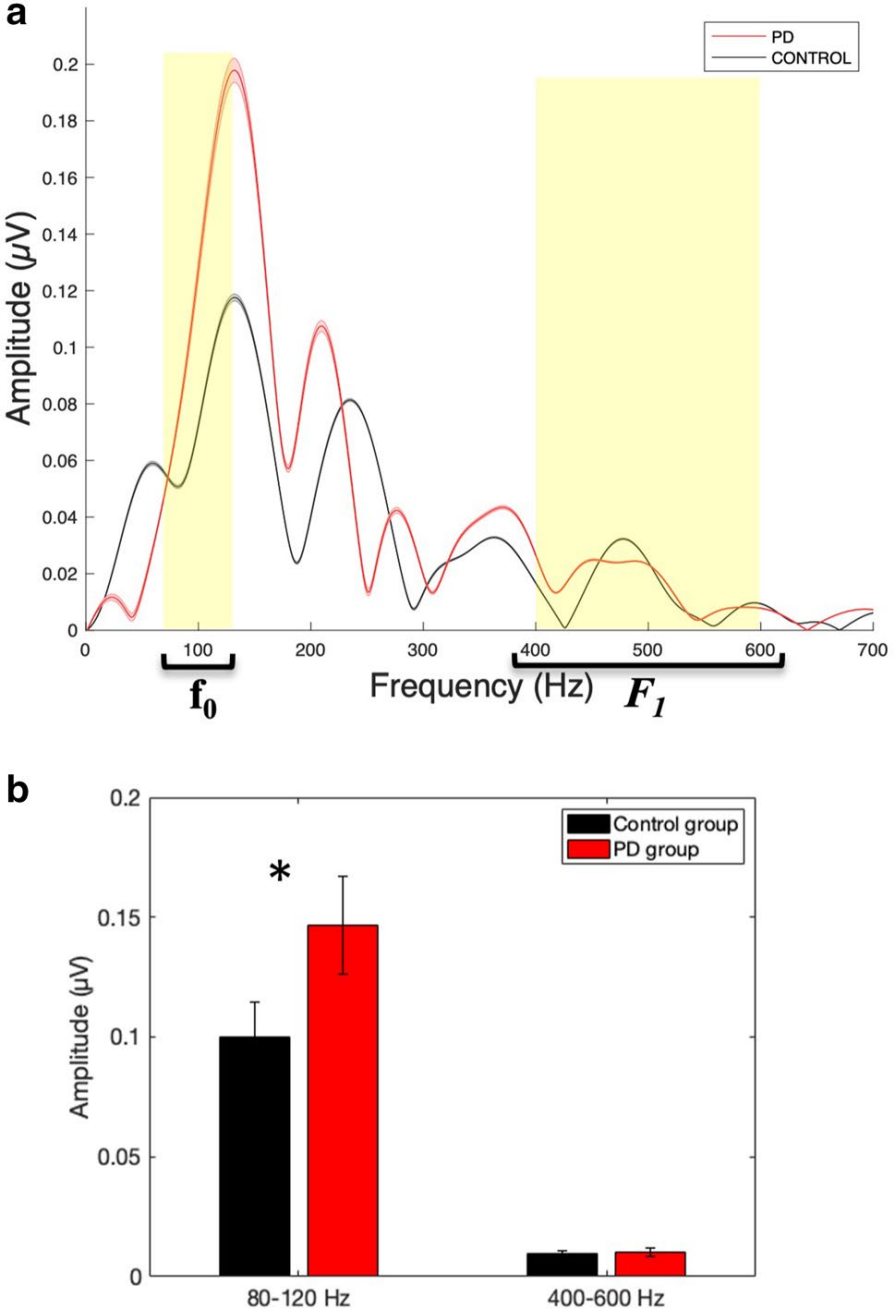
(**a**): Fast Fourier transforms calculated for the vowel part of the average response of individuals with PD (red) and age- and gender-matched control (black) group. (**b**) Bar graphs demonstrating between-group differences in response amplitude corresponding to *f*_*o*_ (80–120 Hz) and *F*_1_ (400–600 Hz) representation of the speech stimuli. The error and shaded error bars represent the standard error. *Note*: *p < 0.01.

Figure 2 shows the averaged FFT (Fig. 2a) and bar graphs of the mean response amplitudes corresponding to *f*_*o*_ and *F*_1_ (Fig. 2b) for the two groups. In the frequency domain, the amplitude of the frequency-following responses during the vowel portion of the stimulus (10–60 ms) for *f*_*o*_ (80–120 Hz) and *F*_1_ (400–600 Hz) were assessed for group differences. Individuals with PD demonstrated larger amplitude FFR responses in the frequency range associated with *f*_*o*_ compared to the control group [*F*(1,28) = 8.51, *p* = 0.007, η_p_^2^ = 0.233; PD group (*Mean *(*M*) = 0.146, *Standard Deviation *(*SD*) = 0.047; *Range* = 0.04–0.23); Control group (*M* = 0.100, *SD* = 0.056; *Range* = 0.04–0.25)]. However, there was no group difference for the frequency range associated with *F*_1_ [*F*(1,28) = 0.002, *p* = 0.966; PD group (*M* = 0.014, *SD* = 0.009; *Range* = 0.01–0.04); Control group (*M* = 0.014, *SD* = 0.006; *Range* = 0.01–0.03)].

### Correlation analysis

To further investigate the relationship between the neurophysiological response to the auditory speech stimuli and the severity of behavioural speech disorders (dysarthria) of PD, we correlated the FFR amplitudes corresponding to *f*_*o*_ and *F*_1_ with the Movement Disorder Society Unified Parkinson’s Disease Rating Scale (MDS-UPDRS) and perceptual dysarthria ratings for each participant with PD. No significant correlation was observed between the amplitudes corresponding to *f*_*o*_ or *F*_1_ and the UPDRS (*f*_*o*_ and MDS-UPDRS: *r*(13) = 0.088, *p* = 0.754; *F*_1_ and MDS-UPDRS: *r*(13) = 0.153, *p* = 0.587) or the perceptual dysarthria scores (*f*_*o*_ and dysarthria score: *r*(13) = − 0.368, *p* = 0.177; *F*_1_ and dysarthria score: *r*(13) = − 0.428, *p* = 0.111).

## Discussion

Our preliminary results are consistent with an enhanced encoding of vocal pitch, evidenced by increased FFR amplitude in the frequency range of 80–120 Hz, in individuals with PD compared to age- and gender-matched control participants. In contrast, we did not find evidence of enhanced encoding within the range of the first formant (400–600 Hz). The increased amplitude of the FFR response is consistent with a selective modulation of the fundamental frequency of the speech stimuli at the level of the brainstem. The brainstem components of the FFR are modulated by the BG through inhibitory and disinhibitory projections to auditory relay areas including the inferior colliculus (IC^30^), with the IC one of the largest generators of the FFR response^32–34^.

Increased encoding of vocal pitch has been observed at the cortical level evidenced by larger auditory evoked potentials (P2 responses) in PD compared to non-PD participants^12^. Since the brainstem FFR can be modulated from the cortex^35^, the specific source of the enhancement (brainstem or cortex) can't be determined from the present results. However, it appears that the increased FFR response in PD participants reflects a reduction in the inhibitory (or tuning) function of the auditory brainstem pathway which enhances the frequency response within the vocal pitch range. In our previous study, PD participants were better able to detect and compensate for pitch shifts compared to the non-PD participants^4^. The current results suggest that the difference in FFR amplitude in the pitch range enhances the salience of the acoustic signal enabling better detection of differences in pitch during listening. In addition, the enhanced feedback signal interacts with the motor output during voice production to produce a stronger response to pitch shifts.

BG damage and its influence on the auditory signal on vocal output during production may be accompanied by an opposite change to the sensitivity of the somatosensory system. Voice production generates auditory as well as somatosensory feedback resulting from the laryngeal vibration, and feedback from both sensory systems contribute to the perception of pitch and loudness^36,37^. For individuals with no history of neurodegenerative disease, vocal fold mucous anesthetization yields a greater compensatory response to auditory feedback alterations in pitch compared to the pitch response without anesthesia^38^, suggesting a trade-off in sensitivity or gain between the two sources of sensory input (somatosensory vs. auditory). Similarly, masking of laryngeal somatosensory feedback by applying low-pass filtered stochastic vibrations to the neck enhances the Lombard response (increased loudness)^37^. In individuals with PD, using air-puff stimulation, it was found that thresholds for detecting laryngeal somatosensory input are increased, consistent with reduced sensitivity of mechanoreceptors in the laryngeal area^39^. As a result, BG damage appears to influence both sensory modalities (auditory and somatosensory) in opposing ways resulting in an increase in sensitivity to pitch-related auditory feedback and a decrease in sensitivity to pitch-related somatosensory feedback.

One consequence of the opposing changes in feedback from the two sensory systems is an imbalance in their contributions to speech production. The addition of the pitch shift would combine with normal feedback to increase the perception of pitch and loudness, while the change in threshold for somatosensory input would result in smaller movements due to the reduced reafferent input. Interestingly, one therapy for remediation of the hypokinetic features in the speech of individuals with PD involves vocal exercises focused on the production of loud speech (Lee Silverman Voice Treatment—LSVT^9,40–43^). The approach generates greater somatosensory feedback from the articulatory and laryngeal systems compared to normal speech. One of the effects of the LSVT is smaller vocal pitch compensations following treatment suggesting a down regulation of the enhanced auditory response^44^. Hence, it is reasonable to suggest that by increasing loudness, the LSVT treatment may act to reset the balance between the feedback systems providing the conditions for more normal speech production and perception.

In our previous work, we also found a reduction in compensation to manipulations of *F*_1_ auditory feedback^10^, and a reduction in *F*_1_ error detection during listening in our PD participants^4^. In the present study there was no evidence of an enhancement or a reduction in the FFR at frequencies in the range of the first formant. The lack of difference in FFR amplitude in the first formant frequency range suggests that the motor and sensory deficits related to *F*_1_ may occur at a higher level than the brainstem. Because formant information is associated with phonetic processing, it more likely involves primary and association levels of the processing stream, which rely on integration of both formant and harmonic information to extract relevant linguistic information. As a result, the influence of BG dysfunction on speech has differential effects related to the processing levels for different components of the speech production (and presumably) the perceptual process.

This study has some shortcomings that should be mentioned. First, most of the individuals with PD in the current study showed mild to moderate severity. It would be of interest to study a greater range of severity in future studies to have a more complete picture of the effect of PD on speech auditory brainstem processing. It is also important to consider other concomitant factors along with PD on the FFR responses, such as amount of musical training or multilingualism (in the case of tonal languages as the second language), as there are reports of the effects of both on FFR responses^26,27^. In order to recruit as many participants as possible, we did not screen for the amount of musical training or multilingualism in the current study, these factors merit attention in future investigations. In addition, based on recent work on different speech subtypes of PD^45,46^, it would be beneficial to assess the relationship of the sensory deficits and different speech subtypes. In the current work, we did not classify different speech subtypes of individuals with PD due to the relatively small sample size. In the future, one can, for example, investigate whether pitch and loudness sensory differences are more frequent in the prosodic subtype of speech deficits in PD as opposed to the phonatory-prosodic or articulatory-prosodic subtypes.

In conclusion, in these preliminary data, we found increased frequency-following neural responses related to *f*_*o*_ during the perception of speech in individuals with PD compared to age- and gender-matched control participants. These findings provide a neural basis for the sensory processing deficits of vocal pitch and loudness at the brainstem level in this population^10,11,13^. Impaired modulation of sensory information at the BG may be one possible factor in the manifestations of speech deficits in individuals with PD.

## Materials and methods

### Ethics statement

This study was approved by the McGill Faculty of Medicine Institutional Review Board, in accordance with principles expressed in the Declaration of Helsinki. Informed written consent was obtained from participants prior to their involvement in the research project.

### Participants

Fifteen patients with Parkinson’s disease (6 female, 9 male; mean age: 65.87 years) and fifteen age- and gender-matched control participants (6 female, 9 male; mean age: 63.13 years) were recruited for this study (same group as our previous study^4,10^). The severity of PD motor symptoms, assessed using the Movement Disorder Society Unified Parkinson’s Disease Rating Scale (MDS-UPDRS; Part III Motor Examination^47^), ranged from mild (a score of 13) to moderate (a score of 48; mean [*M*] ± standard deviation [*SD*] score, 24.79 ± 9.19). Cognitive functioning was assessed using the Montreal Cognitive Assessment (MoCA^48^) and was in the normal range for all individuals with PD (scores > 26). All patients were taking L-dopa in addition to other medication, including dopaminergic and/or anticholinergic drugs. Participants were tested off medication for 12 h. Two participants reported a history of speech therapy focused on increasing speech loudness and intelligibility.

Each participant read aloud the *Rainbow Passage* (a standard speech perceptual passage assessment) in order to carry out a perceptual analysis of dysarthric speech characteristics. A licensed Speech-Language Pathologist rated the speech of participants on 43 perceptual dimensions that span the speech subsystems, including phonatory and articulatory subsystems, using a 7-point scale^1^. Overall, the severity of participants with PD was rated as moderate (2 participants), mild-to-moderate (4 participants), mild (6 participants), and within normal limits (3 participants). Inter-rater agreement was tested between the first rater and a second listener using intraclass correlation (ICC) in order to assess consistency in the ratings of speech perceptual characteristics in individuals with PD. The resulting ICC was in the excellent range, ICC = 0.90^49^, indicating that the raters had a high degree of agreement. We used the perceptual and MDS-UPDRS scores to evaluate any relationship between severity of speech and motor symptoms and the magnitude of the FFR responses.

All participants underwent an audiometric screening and were found to have binaural pure tone hearing thresholds of 40 dB HL or less at 250, 500, 1000, 2000 and 4000 Hz. None of the participants used hearing aids. All participants were native speakers of North-American English. Participants in the control group were healthy with no history of neurological condition.

### Stimulus and recording

A 40 ms (ms) speech syllable, [da], was synthesized at a 20 kHz sampling rate using a Klatt synthesizer^50^. After a 5 ms stop burst, voicing remained constant with a fundamental frequency of 100 Hz, and the first formant frequency of 500 Hz. The [da] stimulus was chosen because it combines transient ([d], the first 10 ms with a 5 ms voice onset time) and periodic ([a]) segments^51^), two acoustic features that have been extensively studied in speech ABR^52^. For each participant, the [da] stimulus was presented 12,000 times with a 50 ms interstimulus-interval. Stimuli were presented in alternating stimulus polarities (i.e., compression and decompression of air molecules of periodic sound waves: positive and negative) to both ears at 80 dB SPL through electromagnetically-shielded insert earphones (Etymotic ER-2) to reduce stimulus and noise artifacts, using a TDT stimulus presentation system (Tucker-Davis Technologies, TDT Inc., Alachua, FL). A vertical montage of four electrodes (left mastoid active, two on the forehead as grounds, and a hairline reference) was used, with all impedances kept under 5 kΩ. Continuous responses were recorded (20 kHz sampling frequency) with ActiABR200 software (BioSemi, Amsterdam, Netherlands). During the recording session (lasting approximately 18 min), participants sat in a comfortable chair in a sound attenuated room.

### Data processing

Electrophysiological responses were band-pass filtered offline in EEGLAB^53^ between 70 and 2000 Hz to maximize signal-to-noise ratio and detection of peaks within the phase-locking limits of the brainstem. The root mean square (RMS) amplitude of the neural responses was used to quantify the overall magnitude of response and pre-stimulus activity. RMS amplitudes were computed for the pre-stimulus period (− 10 to 0 ms) and the response period (0 to 60 ms). A fast Fourier transform (FFT) was performed on a signal window between 10 and 60 ms, corresponding to the voiced portion of the stimulus (Brainstem toolbox^52^). Mean amplitude across frequency ranges corresponding to *f*_*o*_ (80 to 120 Hz) and *F*_1_ (400 to 600 Hz) were calculated for each trial and then averaged across participants for each group. Responses were then averaged over a − 10 to 60 ms window, with stimulus onset occurring at time zero. Any trial with an amplitude greater than 40 µV was considered an artifact and rejected before averaging. We included 11,400 trials of response averages (5700 trials in each polarity) in the analysis after artifact rejection across participants. There were no differences in the number of rejected trials between the two groups [*t*(28) = 0.971, *p* = 2.048]. Responses (0 to 60 ms) were then amplitude baseline-corrected in the pre-stimulus period (− 10 to 0 ms). In a final step, responses from the two stimulus polarities were averaged to minimize the influence of cochlear microphonic and stimulus artifact on the measured response^54^.

### Statistical analysis

Statistical analyses were performed using multivariate analysis of variance (MANOVA) with root mean square (RMS) magnitudes (pre-stimulus and response) and spectral responses (*f*_*o*_ and *F*_1_) as within-subject factors and group (PD vs. control) as between-subject factor. Factor and simple effect sizes were quantified using η_p_^2^ to assess any statistically significant effects, defined as small (0.2–0.3), medium (0.5), and large (> 0.8^55^). Greenhouse–Geisser corrections for unequal variances were applied when necessary. In addition, separate Pearson correlation analyses were performed between FFR amplitudes of *f*_*o*_ or *F*_1_ and perceptual or MDS-UPDRS clinical scores in individuals with PD. This resulted in four correlation analyses. A Bonferroni-adjusted α rate of 0.012 was used.

## Data Availability

Anonymized datasets recorded and analyzed during this study are available from the corresponding author within the limits of participants’ consent.
